# Development of pan-specific antibody against trimethyllysine for protein research

**DOI:** 10.1186/1477-5956-6-2

**Published:** 2008-01-22

**Authors:** Ziqian Liang, Ronald PC Wong, Lin Hong Li, Hesheng Jiang, Hao Xiao, Gang Li

**Affiliations:** 1 Rehabilitation Center of Burns and Plastic Surgery and Medical Research Center, Guangxi Medical University, Nanning, China; 2 Department of Dermatology and Skin Science, Jack Bell Research Centre, Vancouver Coastal Health Research Institute, University of British Columbia, Vancouver, British Columbia, Canada

## Abstract

**Background:**

Trimethylation of the N**ε**-lysine residues in a protein is one of the most important events of posttranslational modifications. Simple methods for rapid detection and isolation of the N**ε**-trimethylated protein species are needed. This report introduces a novel method to prepare the affinity purified antibody specific for the N**ε**-trimethylated lysine (tMeK). The applications of the purified antibody are also reported in this paper.

**Methods:**

We generated the methylated keyhole limpet heomocyanin (KLH) under controlled chemical methylation reaction using CH_3_I and used it as an immunogen to raise anti-methylated lysine antibodies. The tMeK specific antibody was selectively isolated using a two-step affinity chromatography in which the mMeK/dMeK specific antibodies were removed and the tMeK specific antibody was captured. Finally, the eluted anti-tMeK antibody was characterized.

**Results:**

The ELISA results indicated that the antibody reacted only to tMeK but not to mono- and dimethyllysine. Western-blot results showed that the N**ε**-trimethylated proteins were detected in both animal tissue and cultured cells and that the antibody signal could be competitively inhibited with free tMeK.

**Conclusion:**

The specific tMeK antibody we developed is useful for one-step isolation of proteins with N**ε**-trimethyllysine residues and also for the detection, identification and localization of proteins with trimethyllysine residues in the cells.

## Background

Similar to protein phosphorylation and acetylation, protein methylation is one of the most important post-translational modifications in regulating protein functions. Perhaps, histone is the best known target for protein methylation. Histone lysine methylation plays an important role in the regulation of chromatin structure, gene expression and DNA damage [1,2]. Histones and p53 could be enzymatically methylated by a family of protein lysine methyltransferases [3,4] and demethylated enzymatically by a family of demethylases [5,6]. The single lysine residue could exist as mono-, di-, tri-methylated and unmethylated forms with different functional consequences [7]. Trimethylation of a specific lysine residue in histone H3 is reported to be associated with inactive X chromosome [5,8,9].

Most of the reports on protein methylation are related to histone methylation and a few of them related to the p53 methylation [10]. Other methylated proteins are still largely unknown. As protein methylation may be associated with embryonic development and diseases [1], the proteomic survey of the methylated protein patterns in different developmental stages and human diseases become an important area in epigenetic research. In this paper, we report a novel method to generate and purify a pan-specific, N**ε**-trimethyllysine antibody (anti-tMeK), which could be used as a simple tool for the study of protein trimethylation profiles. We generated the methylated keyhole limpet heomocyanin (KLH) under controlled chemical methylation reaction using CH_3_I and used it as an immunogen to raise anti-methylated lysine antibodies. The tMeK specific antibody was selectively isolated using a two-step affinity chromatography in which the mMeK/dMeK specific antibodies were removed and the tMeK specific antibody was captured. Finally, the eluted anti-tMeK antibody was characterized and we demonstrated that the anti-tMeK antibody could be used as a functional tool for the detection of the trimethylated proteins using Western blot and immunofluorescence and isolation of trimethylated proteins using immunoprecipitation.

## Results

### Purification and ELISA characterization

**Figure 1:**
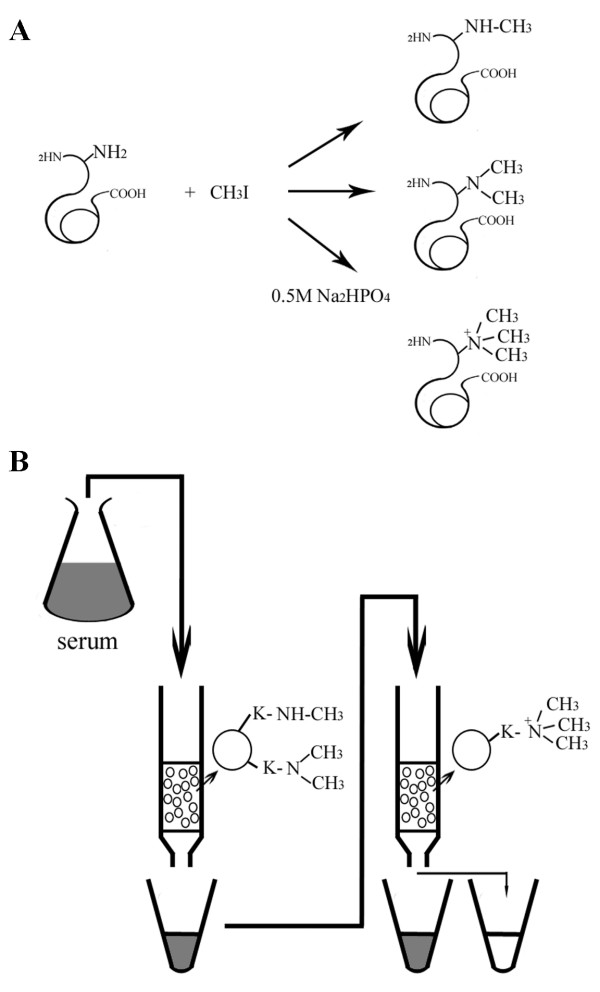
Generation of anti-trimethyllysine antibody. **A**. The reaction scheme for chemical synthesis of KLH with trimethyllysine residues. **B**. The scheme for strategic affinity purification of the trimethyllysine-specific antibody. The antibody in serum cross-reacted to monomethyllysine and dimethyllysine is removed by the mMeK/dMeK affinity column. The remaining trimethyllysine-specific antibody in the serum is further purified using tMeK affinity column.

Ideally, a trimethyllysine-specific antibody (anti-tMeK) should be highly specific and has strong affinity to trimethyllysine (tMeK) but not to monomethyllysine (mMeK) and dimethyllysine (dMeK). The rationale of developing such an antibody is to use a synthetic trimethylated protein, in which, the N**ε**-amino group of the lysine side chain was trimethylated, as an immunogen. Methylation of the ε-amine groups of a protein using iodomethane (CH_3_I) generates mono-, di- and trimethylated lysine residues in a protein (Figure 1a). The yield for trimethylated species was increased by performing the reaction at higher pH and in prolonged reaction time (see Materials and Methods). The highly immunogenic protein, KLH, was used for the methylation reaction. The dialyzed methylated KLH was immunized to the rabbit to generate anti-methyllysine antibodies. After 60 days from initial immunization, immune serum was prepared and ELISA test was carried out to test the titer. The serum has 50% maximum OD titer at 1:50,000 to tMeK-BSA conjugates, approximately 1:2,000 and 1:10,000 to mMeK-BSA and dMeK-BSA conjugates respectively (data not shown). The methylated KLH was synthesized while the mMeK, dMeK and tMeK used in ELISA were purchased commercially. The primary ELSIA results indicated that the anti-methylated KLH immune serum cross-reacted with each form of the methylated lysine peptides.

The rabbits immunized with methylated KLH contained specific antibodies against all the methylated species. The strategy for isolating and purifying the tMeK-specific antibody is illustrated in Figure 1b. The immune serum was initially passed through a mMeK/dMeK affinity column. The antibodies bound to the column was eluted and determined to be about 10 mg. SDS-PAGE staining suggested the eluted proteins were mainly IgG with over 90% purity (data not shown). The yield for mMeK/dMeK antibodies was approximately 50 μg/mL of serum.

After removal of the mMeK/dMeK antibodies, the immune serum flowed through the mMeK/dMeK affinity column was then passed through the tMeK affinity column. The antibody bound to the tMeK column was eluted and determined to be approximately 50 mg. SDS-PAGE staining indicated that the IgG purity is over 90% (data not shown). The yield for tMeK-specific antibodies was approximately 250 μg/mL.

**Figure 2:**
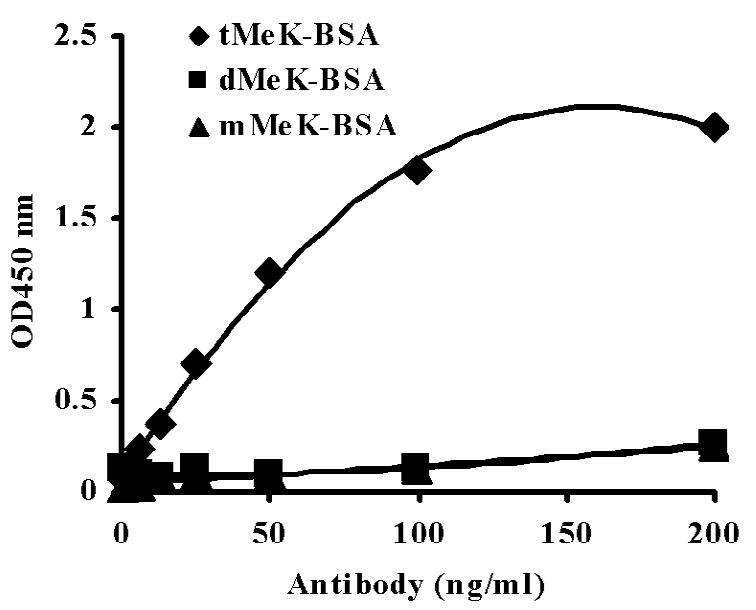
The specificity of the tMeK-purified antibody in an ELSIA test. The ELISA reaction of the purified tMeK-specific antibody was carried out with immobilized tMek-BSA, mMeK-BSA, or dMeK-BSA.

The specificity of the purified anti-trimethyllysine to immobilized mMeK-, dMeK- and tMeK-BSA was determined by indirect ELISA (Figure 2). BSA is used here to facilitate the immobilization of the small methyllysine molecule onto the plastic surface of the micro plate. The chemical reaction for the preparation of three forms of methyllysine-BSA conjugates is identical. The primary antibody dilution buffer also contains 1% of BSA. The ELISA results showed that the tMeK-specific antibody did not react well with both mMeK and dMeK-BSA but strongly to tMeK.

### Immunoblotting detection and immunoprecipitation of the proteins with Nε-trimethyllysine residues in cell culture and animal tissue

**Figure 3:**
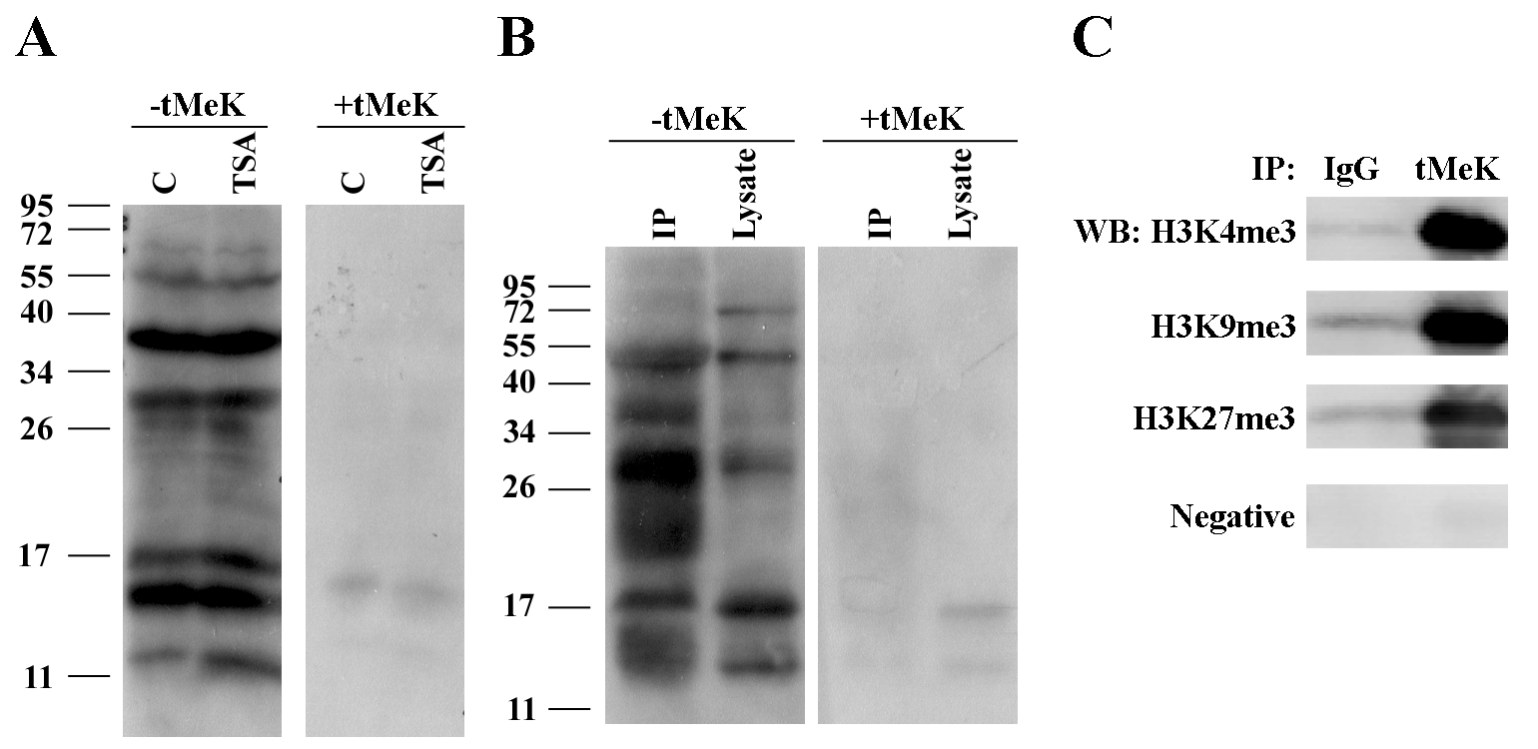
Western blot and immunoprecipitation analysis of proteins from human melanoma cells using the anti-tMeK antibody. **A**. Cells treated with or without TSA were lysed for Western blot (50 μg/lane) using the trimethyllysine-purified antibody, with and without the presence of free tMeK. **B**. Western blot analysis of the anti-tMeK immunoprecipitated proteins from the crude lysate of the mouse spleen using anti-tMeK HRP conjugates (0.25 μg/ml). The signal was competitively inhibited by the free tMeK (10 μg/ml). **C**. Western blot analysis of immunopreciptates by IgG control or anti-tMek antibody with H3K4me3, H3K9me3, or H3K27me3 antibodies.

Figure 3a showed that the anti-tMeK antibody recognized various proteins in the human melanoma cells. Those protein bands recognized by the anti-tMeK antibody could be completely inhibited by the free tMeK suggesting that the antibody is Nε-trimethyllysine-specific and the protein bands recognized by the anti-tMeK are trimethylated.

There are 7 major protein bands (14, 17, 19, 30, 32, 40, and 54 kD) recognized by anti-tMeK antibody according to the Western blot (Figure 3a). The strongest bands recognized by tMeK-specific antibody were 17 kD and 40 kD. The film exposure time for the blot was less than 1 min. Some of the proteins with lower expression recognized by the antibody may not be revealed under such conditions. Since lysine residues of histones can also be acetylated, we tested if the histone deacetylase inhibitor trichostatin A (TSA) would have an effect on trimethylation of cellular proteins. Our data showed that TSA increased the trimethylation level, particularly for the 14 kD and 18 kD proteins.

In the mouse spleen lysate, approximately 5 major protein bands were recognized by the anti-tMeK antibody. The methylated protein pattern is somewhat similar to melanoma cell lysate. The major bands recognized are 14, 17, 30, 54, and 70 kD (Figure 3b). The protein bands recognized by the antibody could also be completely inhibited by free tMeK.

Immunoprecipitation in undenatured condition is important for protein isolation and purification. Approximately 200 μg of anti-tMeK antibody was utilized to isolate possible trimethylated proteins. Using the anti-tMeK HRP conjugates for the Western blot analysis of the anti-tMeK immunoprecipitated proteins, the massive interference of IgG bands produced by the secondary anti-rabbit IgG HRP could be eliminated. No IgG signal was observed in the competitive inhibition by tMeK BSA (Figure 3b). The Western blot results however, showed different pattern of proteins. In the crude lysate, only 5 major protein bands were recognized by the anti-tMeK HRP while the signal in the crude lysate was significantly enriched in the IP sample. However, the 70 kD proteins detected in crude lysate could not be isolated by anti-tMeK antibody (Figure 3b). In the IP sample, anti-tMeK could capture more than 6 protein bands with 14–16, 17, 20–23, 30, 40, and 54 kD. The protein band at 70 kD was only captured by tMeK in crude lysate. The 20–23 kD, 30 kD proteins were significantly enriched while the 20–23 kD bands were not visible in the crude lysates. Apparently all the IP protein bands were blocked with the presence of free tMeK molecule, suggesting that the IP proteins contained tMeK-residues.

To confirm whether the proteins immunoprecipitated by the antibody we generated are indeed trimethylated, we used three well established site-specific anti-trimethyllysine antibodies against histone H3 (H3K4me3, H3K9me3, H3K27me3) for Western blotting. Figure 3c shows that all three trimethylated species of histone H3 could be detected in the proteins immunoprecipitated with our antibody.

### Immunofluorescent staining

**Figure 4:**
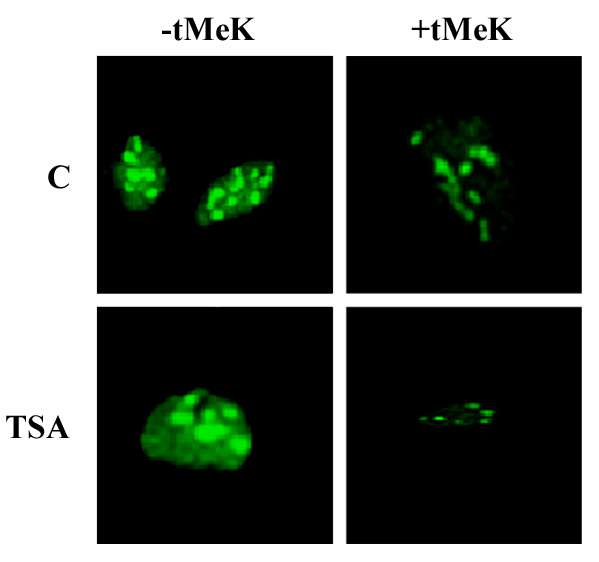
Immunofluorescence staining of the human melanoma cells using tMeK-specific antibody. Cells treated with or without TSA (200 ng/ml) for 16 h were fixed with formaldehyde and permeabilized with 1% triton X-100. The cells were stained with 10 μg/ml anti-tMeK-FITC antibody with and without the presence of free tMeK (10 μg/ml). The image was captured with the Zeiss fluorescence microscope equipped with Northern Eclipse imaging software with an exposure time of 100 ms. The picture shows the representative images of the staining.

Using the human melanoma cells as working model, the FITC conjugated anti-tMeK antibody could strongly stain the nuclei of the cell and weakly in the cytoplasm. Brighter spots of the staining could also be observed in the nuclei (Figure 4). The fluorescent staining signal could be significantly inhibited with the presence of the tMeK, suggesting that staining signal is trimethyllysine-specific.

## Discussion and Conclusion

Protein methylation is an important event of post-translational modification. The goal of proteomic studies is to reveal that how the protein modification plays its role in gene expression, protein interaction and signal transduction. To understand the roles of protein methylation, simple and rapid methods for detection, identification, isolation and localization of the methylated proteins is needed. Development of an antibody specific for the methylated proteins is one of the important aspects to facilitate the proteomic survey of the protein methylation. The pan-specific antibodies against mMeK and dMeK have been commercially available. The purpose of this research is to develop a specific antibody against tMeK which could be applied to the proteomic survey and to the basic research of bioscience.

In our previous report [11], we used modified protein as immunogen and antibody against single modified amino acid could be raised and isolated through specific immunoaffinity chromatography. To produce an antibody against single molecule of trimethyllysine, a trimethylated protein may be the best choice as immunogen. Enzymatic synthesis of trimethylated protein may not be a good choice for generation of such immunogen as the enzyme reaction is highly site-specific. Chemically trimethylated proteins may produce desired epitopes in an immunogenic protein. Reductive methylation of proteins using formaldehyde followed by mild reduction with cyanoborohydride makes this possible. However, the products mainly consist of mono- and di-methylated lysine instead of trimethyllysine [12]. We therefore sought to use CH_3_I to methylate the Nε-amino group of lysine residues in a protein at high pH (pH > 9). Primary and secondary amines are readily methylated under this condition. The Nε-methylation products are controlled by the pH, time and temperature of the reactions. A conventional high immunogenic protein KLH was methylated with CH_3_I at pH > 9, 35°C for more than 6 h. Presumably, the higher in pH and temperature, longer reaction time, the more trimethylamine species will be generated. The resulting methylated immunogen may yield more trimethylated protein specific antibodies.

The initial ELISA titration of the crude serum indicated that the population of the antibodies against KLH methylated by CH_3_I has higher affinity for the trimethyllysine (tMeK). Subsequently, the immunoaffinity chromatography also demonstrated that our rationale is correct. Approximately 80% (50 mg) of the methyllysine specific antibodies specifically bind to tMeK, while only about 20% (10 mg) of the antibodies bind to mMeK/dMeK column. The ELISA data (Figure 2) showed that the antibody purified with tMeK (anti-MeK) only recognized the tMeK-BSA, not mMeK- or dMeK-BSA, suggesting that the anti-tMeK antibody is highly specific.

The ELSIA data provide us the information that this antibody is specific for tMeK not mMeK or dMeK. Using human melanoma cell culture as working model, Western blot analysis indicated that multiple proteins with different molecular weight range from 14 kD to 54 kD could easily be detected with the anti-tMeK-specific antibody (Figure 3a). Similar finding was also observed when the antibody was applied to the protein extract of the mouse tissue extract (Figure 3b). The protein bands recognized by this tMeK-specific antibody could be completely inhibited by the presence of free tMeK, suggesting the antibody could be specifically used for detection of the proteins with tMeK residues. The best known trimethylated proteins are histones, particularly the histone H3 [2,13,14]. The Western blot results of both cultured cells and animal tissue indicated that the 17 kD band may correspond to histone H3. In addition, using the well established site-specific anti-trimethyllysine antibodies against histone H3 confirmed the presence of histone H3 in the immunoprecipitates isolated with our antibody. There are many protein bands recognized by anti-tMeK not only in Western blot but also in immunoprecipitation. The results imply that many other trimethylated proteins remain unidentified.

In order to study the trimethylation profile of the proteins, methods for rapid isolation of the trimethylated species is needed. Immunoaffinity chromatography or IP with anti-tMeK is one of the most efficient methods for this purpose. Immunoprecipitation studies (Figure 3b &3c) showed that this tMeK specific antibody could capture the trimethylated species efficiently. Most of the trimethylated proteins in the crude lysate recognized in the Western blot detection system could also be specifically isolated from the crude lysate by immunoprecipitation and some of them could be significantly enriched by immunoprecipitation implying the potential use of this anti-tMeK antibody in isolating trimethylated proteins for proteomics study.

Immunofluorsecent staining of human melanoma cells with this anti-tMeK showed strong staining in the nuclei (histone associated) and weak signal in the cytosol. The immunofluorescent signal of the antibody was blocked by free tMeK, suggesting the antibody staining signal is highly specific for tMeK. The immunofluorscent staining result correlates well with the fact that the lysine residues of histones could be trimethylated at multiple sites and the expression level of histones is usually high and stable. The bright spots of the nuclear immunofluorescent staining could be explained by the fact that some histones associated with the chromosome maybe heavily trimethylated [8]. Those spots may also be the heterochromatin in silenced region of the genome [4].

Protein methylation is one of the important post-translational modifications. The role of the protein methylation in protein functions is not completely understood. Proteomic studies of the protein methylation pattern in different cell cycle stages and disease states, in response to gene expression regulation and drug treatment would be interesting. Using the tMeK-specific antibody for Western blot, the trimethylated protein patterns could be detected and identified. Using immunoaffinity-capture techniques, trimethylated proteins could be specifically isolated by this tMeK-specific antibody and further identified through peptide sequence analysis, Western blot or ELISA screening, microarray screening and mass spectrometry database screening. Presumably, this antibody can also selectively capture the trimethylated peptide fragments as a result of the protease digestion of purified proteins. Therefore, this could facilitate the proteomic survey of the trimethylated proteins and identify the specific site of trimethylation. Similar approach has been reported using acetyllysine-specific antibody to identify the acetylated proteins in the cell through the immunoaffinity-capture technique and MS analysis [15].

In summary, CH_3_I is a good choice for chemical trimethylation of proteins as such immunogen yields more antibodies against tMeK than the antibodies against mMeK and dMeK. Single molecule of methylated lysine could be used as immobilized affinity ligand to selectively purify the specific population of the polyclonal antibody against each specific form of Nε-methyllysine. The tMeK specific antibody prepared using this method is proven to be useful for detection, identification, isolation and localization of the Nε-trimethylated proteins.

## Methods

### Methylation of KLH

All reagents except mentioned were purchased from Sigma-Aldrich (St. Louis, MO). 25 mg of KLH was dissolved in 10 ml of 0.5 M Na_2_HPO_4_, then 10 μl of the iodomethane solution was added every 30 min at 35°C for 2 h. The reaction was incubated at 35°C for another 4 h. Saturated Na_2_CO_3 _was added to maintain the pH of the reaction greater than pH 9. The reaction was stopped with 50 μl of the 2-mercaptoethanol at room temperature for 30 min. The methylated KLH was then dialyzed against PBS 4 times at 2-h intervals. After dialysis, the total protein concentration was determined using the bicinchoninic acid kit (Sigma) as described by the supplier.

### Immunization

The methylated KLH (0.5 mg/rabbit) was immunized to three rabbits (New Zealand species) with complete Freund adjuvant. The rabbits were boost with 0.25 mg methlylated KLH each with incomplete Freund adjuvant in 14 days, 35, and 56 days after the initial immunization, respectively. The rabbits were bled for serum preparation 60 days after the initial immunization.

### ELISA

5 mg of mMeK, dMeK and tMeK were dissolved in 1 ml of 0.1 M Na_2_CO_3_. 5 μl of 70% glutaraldehyde was added at room temperature for 1 min; then all the reaction mixture was transferred to 10 ml of BSA (2 mg/ml in 0.1 M Na_2_CO_3_) in a 50 ml centrifuge tube. The reaction was incubated at 35°C for 4 h then cooled to 4°C. 10 mg of solid sodium cyanoborohydride was added to each reaction. Each reaction mixture was then dialyzed against 500 ml of PBS for four times at 2-h interval. The conjugates were stored in aliqouts at 4°C before use.

mMeK, dMeK and tMeK-BSA conjugates were diluted to 20 μg/ml in 0.1 M Na_2_CO_3_. The ELISA plate was coated with 100 μl of methylated BSA conjugates in each well and was incubated at room temperature for overnight. The plate was washed with PBST (PBS containing 0.04% Tween 20) and blocked with 100 μl/well of 2.5% BSA at room temperature for 2 h. The plate was washed with PBST for three times.

The serum was titrated from 1:1,000 in each methyllysine-BSA plate in 2-fold serial dilution. The purified anti-tMeK antibody was titrated in plates coated with mMeK-, dMeK- and tMeK-BSA. Samples were run in triplicates. The starting concentration of the anti-tMeK is 250 ng/ml in PBST containing 1% BSA and was diluted in a 2-fold serial dilution. The primary antibody was incubated for 60 min and washed with PBST for 4 times. Then, anti-rabbit IgG HRP conjugates (0.25 μg/ml) in PBST containing 1% BSA were added and the reaction was incubated at room temperature for 30 min. After washing with PBST for 4 times, 100 μl/well of HRP substrate TMB was added for color development. The color reaction was stopped in 20 min with 20 μl of 1N HCl and absorbance at OD450 nm was measured.

### Affinity purification of Anti-tMeK antibody

10 ml of agarose beads (Sepharose CL4B) was suspended in 25 ml of 0.1 M acetate buffer (pH 6.5), and 200 mg of the sodium periodate was added at 35°C for 2 h to oxidize the agarose to yield aldehyde functional groups. The beads were washed with the 400 ml of the acetate buffer (pH4.5) then with 100 ml of distilled water. 5 mg of mMeK and dMeK and 15 mg of tMeK were dissolved in 1 ml of 0.1 M Na_2_CO_3_. The coupling reaction was performed by adding mMeK, dMeK and tMek, respectively, into the oxidized agarose (W/V = 1/2) at 35°C for 2 h then cool to 4°C. Then, 100 mg of the solid sodium cyanoborohydride was added to each reaction and incubated overnight. The beads were washed sequentially with 100 ml of distilled water and stored at 4°C.

The scheme for the affinity purification of tMeK-specific antibody was illustrated in Figure 1. 10 ml of mMeK and 10 ml of dMeK immobilized agarose (20 ml in total) were packed into one glass column (2.5 × 10 cm) and 30 ml of the tMeK agarose into another one glass column (2.5 × 10 cm). The column was washed with 200 ml PBST then 10 ml control blank rabbit serum was allowed to pass through each column and washed with 100 ml of PBST and 50 ml of the 3% acetic acid. The columns were then equilibrated with 50 ml of PBS.

200 ml of the immune serum was initially passed through the 20 ml mMeK/dMeK column at a flow rate of 5 ml/min. The column was initially washed with 200 ml of PBST then with 50 ml of 2 M NaCl. The antibody was eluted from the column with 3% acetic acid and collected on ice. After 5 ml void volume, 25 ml of eluent was collected and 3 g of ammonia sulfate was added per 10 ml of eluent to precipitate the eluted antibody. The solution was continuously stirred until all ammonia sulfate was dissolved. The solution was placed at 4°C overnight and the antibody was precipitated by centrifugation. The column was washed with PBST again then equilibrated with PBS. The flow through was then passed through the same column again until all the mMeK/dMeK specific antibodies were removed by the column.

The mMeK/dMeK column adsorbed flow through was then passed through a 30 ml tMeK column at a flow rate of 5 ml/min. The column was initially washed with 250 ml of PBST then with 100 ml of 2 M NaCl. The bound antibody was eluted with 50 ml 3% acetic acid. After 8 ml void volume, 35 ml of eluent was collected, and 3 g of ammonia sulfate/10 ml of eluent was added immediately to precipitate the eluted antibody. The tube was incubated at 4°C overnight and the antibody was precipitated by centrifugation.

The precipitated antibody is dialyzed against PBS and subjected to SDS-PAGE characterization and protein determination using human IgG as a standard.

### Antibody conjugation

20 mg of HRP was dissolved in 4 ml of citrate buffer (pH 6.5) and 5 mg of sodium periodate was added and incubated at 35°C for 30 min. The reaction was stopped by passing the mixture through a 15 ml G25 Sephadex column. The brown color fraction of HRP was collected and stored at 4°C.

2 mg of the purified antibody was added in 0.5 ml of 0.1 M Na_2_CO_3 _and mixed with 1 mg of the activated HRP at 35°C for 15 min then cooled to 4°C. 1 mg of sodium borohydride was added and incubated overnight. Sodium borohydride was removed by passing the solution through 15 ml of G25 Sephadex column equilibrated with PBS. The brown HRP fraction was collected and was stored in presence of 50% glycerol and 10 mg/ml BSA -20°C.

2 mg of the purified antibody was added in 0.5 ml of 0.1 M Na_2_CO_3_. 1 mg of the FITC was dissolved in 50 μl of DMSO. 10 μl of the dissolved FITC was added to the antibody solution, vortexed and incubated at room temperature for 30 min. The reaction was stopped by adding 10 μl of saturated Tris and then passed though a 15 ml G25 Sephadex column equilibrated with PBS. The yellow FITC-labeled antibody fraction was collected and stored in presence of 50% glycerol and 10 mg/ml BSA at -20°C.

### Cell culture

Melanoma MMRU cells, a gift of Dr. R. Byers, Boston University, were cultured with Dulbecco's Modified Eagle's Medium (Gibco) supplemented with 10% fetal bovine serum (Hyclone), 100 units/ml penicillin and 100 μg/ml streptomycin in 5% CO_2 _atmosphere at 37°C. For trichostatin A (TSA) treatment, MMRU cells were treated with 200 ng/ml TSA for 16 h.

### Immunoprecipitation

A mouse spleen was crunched and homogenized with lysis buffer (50 mM Tris-HCl [pH 8.0], 150 mM NaCl, 0.02% sodium azide, 0.1% SDS, 1% NP-40, 0.5% sodium deoxycholate, 100 μg/ml phenylmethylsulphonyl fluoride, 1× protease inhibitor), and the lysate was centrifuged at 12,000 × *g *for 5 min. The supernatant was used for both IP and western blot. 100 μl of Protein A was packed in a mini-column and anti-tMeK antibody (200 μg) in PBS was pre-immobilized to the Protein A beads by passing the antibody through the Protein A bead column 4 times and washed with PBST. The crude lysate of the mouse spleen (5 mg) was passed through the anti-tMeK pre-fixed Protein A beads once, washed with PBST extensively, eluted with 200 μl of hot 2× SDS-loading buffer and the eluent was collected.

### Western blot

Whole cell lysate was prepared from MMRU cells by lyzing the cells in lysis buffer and sonicated. Cell debris was removed by centrifugation at 12,000 × *g *for 10 min and the supernatant was recovered. Protein concentration was quantified by Protein Assay (Bio-Rad, Hercules, CA). 50 μg of proteins and 20 μl of IP sample were loaded for SDS-PAGE, transferred to polyvinylidene difluoride membrane, blocked in 5% BSA in PBST and probed with HRP-conjugated anti-tMeK antibody at 1:1,000 dilution with or without 10 μg/ml tMeK. Signals were developed by ECL system (Amersham, Piscataway, NJ) and visualized by autoradiography.

### Immunofluorescent staining

Subconfluent MMRU cells seeded in a 6-well plate with or without HDAC inhibitor treatment were fixed and permeabilized with 1% formaldehyde and 0.5% Triton X-100 for 10 min and blocked with 10 mg/ml BSA for 1 h at room temperature. Cells were stained with FITC-conjugated anti-tMeK antibody (10 μg/ml) with or without 10 μg/ml tMeK for 1 h at room temperature. Photos were taken with Zeiss fluorescence microscope equipped with Northern Eclipse imaging software with an exposure time of 100 ms.

## Competing interests

The author(s) declare that they have no competing interests.

## Authors' contributions

ZL performed antibody production, purification and immunoprecipitation. RPCW carried out tissue culture, immunofluorescent staining, immunoblotting, and manuscript preparation. LHL and HJ participated in the antibody preparation. HX and GL consolidated the study design, data analysis and finalized the manuscript. All authors have read and approved the final manuscript.
